# Laparoscopic vs. open feeding jejunostomy insertion in oesophagogastric cancer

**DOI:** 10.1186/s12893-021-01318-9

**Published:** 2021-10-13

**Authors:** Sotiris Mastoridis, Giada Bracalente, Christine-Bianca Hanganu, Michela Neccia, Antonio Giuliani, Richard Gillies, Robert Marshall, Nicholas Maynard, Bruno Sgromo

**Affiliations:** 1 Oxford Oesophagogastric Centre, Churchill Hospital, Oxford University Hospitals NHS Foundation Trust, Old Road, Oxford, OX3 7LE UK; 2 University of L’Aquila Medical School, L’Aquila, Italy; 3 University of Cambridge Medical School, Cambridge, UK

**Keywords:** Laparoscopic, Feeding, Jejunostomy, Oesophagogastric, Cancer

## Abstract

**Background:**

Jejunal feeding is an invaluable method by which to improve the nutritional status of patients undergoing neoadjuvant and surgical treatment of oesophageal malignancies. However, the insertion of a feeding jejunostomy can cause significant postoperative morbidity. The aim of this study is to compare the outcomes of patients undergoing placement of feeding jejunostomy by conventional laparotomy with an alternative laparoscopic approach.

**Methods:**

A retrospective review of data prospectively collected at the Oxford Oesophagogastric Centre between August 2017 and July 2019 was performed including consecutive patients undergoing feeding jejunostomy insertion.

**Results:**

In the study period, 157 patients underwent jejunostomy insertion in the context of oesophageal cancer therapy, 126 (80%) by open technique and 31 (20%) laparoscopic. Pre-operative demographic and nutritional characteristics were broadly similar between groups. In the early postoperative period jejunostomy-associated complications were noted in 54 cases (34.4%) and were significantly more common among those undergoing open as compared with laparoscopic insertion (38.1% vs. 19.3%, P = 0.049). Furthermore, major complications were more common among those undergoing open insertion, whether as a stand-alone or at the time of staging laparoscopy (n = 11/71), as compared with insertion at the time of oesophagectomy (n = 3/86, P = 0.011).

**Conclusions:**

This report represents the largest to our knowledge single-centre comparison of open vs. laparoscopic jejunostomy insertion in patients undergoing oesophagectomy in the treatment of gastroesophageal malignancy. We conclude that the laparoscopic jejunostomy insertion technique described represents a safe and effective approach to enteral access which may offer superior outcomes to conventional open procedures.

**Supplementary Information:**

The online version contains supplementary material available at 10.1186/s12893-021-01318-9.

## Background

Oesophageal cancer is the eighth most common cancer worldwide [1]. It is a highly lethal condition, leading to over 400,000 deaths annually [2]. The incidence of weight loss in patients with oesophageal cancer is among the highest of all cancer types. The majority of patients with oesophageal cancer will have experienced a significant degree of weight loss by the time of diagnosis and will have complex nutritional needs [3]. The effect of such weight loss on patient performance status can be detrimental to the delivery of gold-standard multimodal treatment strategies and, consequently, can adversely impact survival outcomes. The likelihood of encountering severe dose-limiting toxicity is substantially greater among individuals with nutritional deficiency who undergo chemotherapy [4]. It is evident, therefore, that the nutritional status of patients with oesophageal cancer should be carefully considered during the diagnostic and staging phases; and routine nutritional assessment should form a central component of the multimodal treatment of patients [5].

In their systematic review and meta-analysis of randomized controlled trials, Mazaki and colleagues demonstrated that, when compared to parenteral nutrition, enteral nutrition was associated with fewer infectious complications, anastomotic leaks, intra-abdominal abscesses, overall complications, and decreased length of hospital stay [6]. Feeding jejunostomy is the most commonly employed approach to enteral nutrition in patients undergoing treatment for oesophageal malignancy, given that sparing of the stomach is necessary for its potential use as a conduit to replace the resected oesophagus.

Jejunostomy insertion can be performed open via laparotomy, laparoscopically, or by percutaneous techniques assisted either endoscopically or using other imaging modalities. In patients with severe dysphagia or with significant weight loss (≥ 10%) at presentation, a jejunostomy is usually placed at the time of the staging laparoscopy or as a stand-alone procedure. The goal is to support the patient’s nutritional status and hydration during neo-adjuvant treatment. When a jejunostomy is not indicated before neo-adjuvant treatment it is usually placed at the time of oesophagectomy to provide fluids and nutrition if the oral route is compromised, for instance in the case of anastomotic leak. Given the variation in timing and technique of jejunostomy insertion, the aim of this study is to compare outcomes of subjects undergoing open and laparoscopic jejunostomy insertion in the management of oesophageal malignancy.

## Methods

Retrospective review was performed of databases prospectively compiled between August 2017 and July 2019 at the Churchill Hospital, Oxford, UK. The indication for jejunostomy was decided by multidisciplinary team discussion involving surgeons, oncologists, gastroenterologists, and dietitians according to the severity of dysphagia, weight loss, and nutritional status. Demographic data and baseline patient characteristics including age, sex, body mass, preoperative serum albumin, performance status (Eastern Cooperative Oncology Group, ECOG), American Society of Anaesthesiologists physical status classification (ASA grade), TNM staging, relevant comoborbidities, and oesophagogastric tumour histology were reviewed. All jejunostomy procedure-related complications were collated and classified in accordance with Clavien-Dindo (CD) scoring, whereby minor complications are those scoring 2 or less and major complications constitute those scoring 3 or more [7].

### Surgical techniques

For both the open and laparoscopic techniques described, a 9 Fr feeding jejunostomy tube kit was used (Freka^®^ Surgical Jejunostomy Set ENFit^®^, Fresenius Kabi). In instances where feeding jejunostomy placement was performed at the time of oesophagogastrectomy, laparoscopic insertion was performed where the abdominal phase of the resectional procedure was performed minimally invasively. The decision as to whether jejunostomy was performed laparoscopically or open at SP was determined primarily by surgeon preference. Open insertion was performed via a midline laparotomy employing the Witzel technique—the most common method of jejunostomy creation [8]. The duodenojejunal (DJ) flexure was identified and a loop of jejunum selected and confirmed to reach the abdominal without excess tension. The jejunostomy tube was introduced into the jejunum using the dedicated kit through a purse string suture made with absorbable monofilament on the small bowel. A 2 cm submucosal tunnel was created with the introducer and subsequently a short Witzel tunnel made with 3/0 absorbable monofilament to prevent leak of enteric content. The small bowel was secured to the abdominal wall with parachuting sutures, and the jejunostomy secured to the skin with the flange provided using non absorbable braided or monofilament sutures. Where open jejunostomy was performed concurrently with open oesophagectomy, the jejunostomy was introduced via the established abdominal incision.

**Fig. 1:**
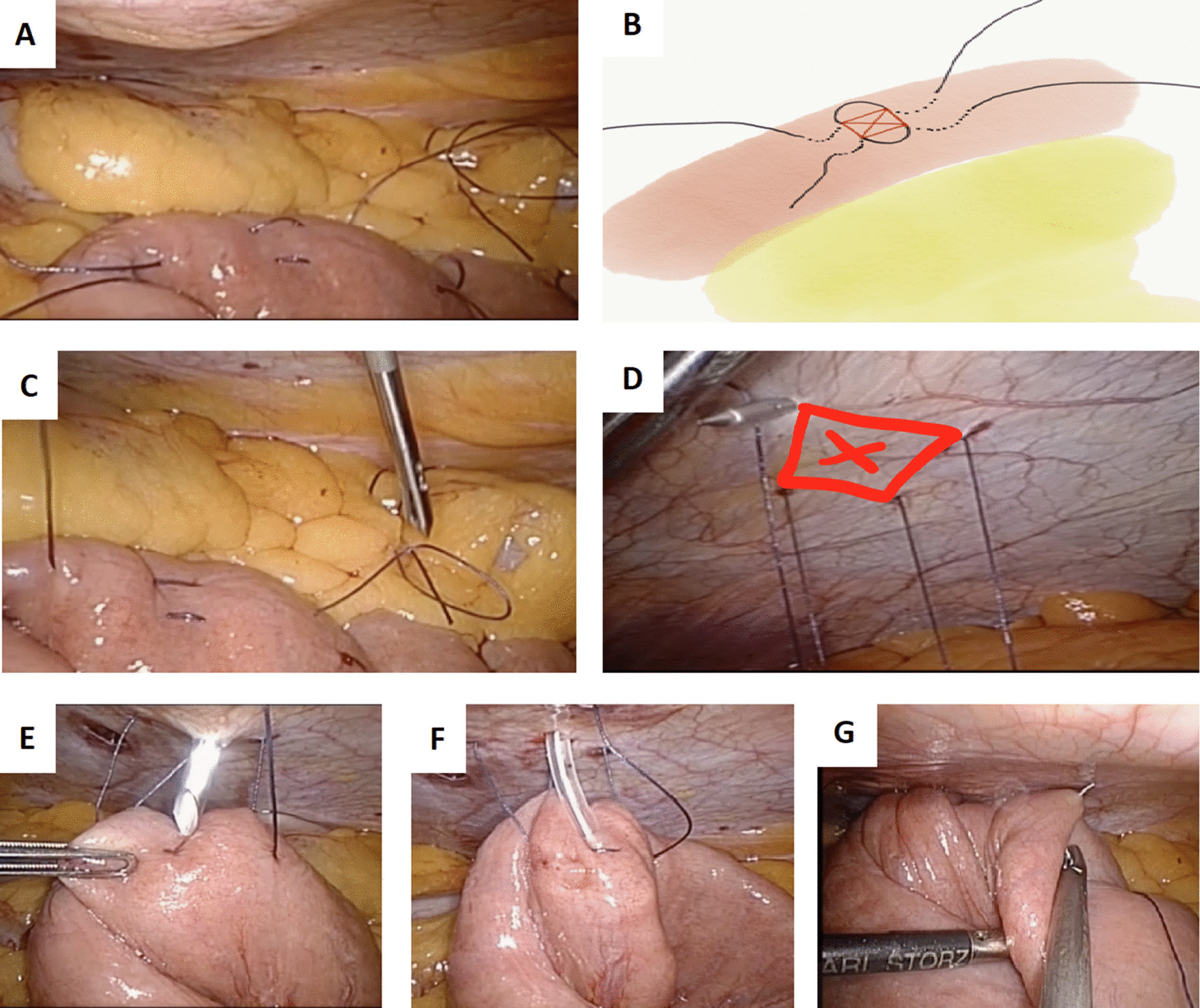
Laparoscopic Jejunostomy technique. **A** Two jejunal sutures inserted in ‘W’ configuration leaving an approximately 1 cm^2^ box target as shown in red in schematic representation (**B**). **C** Endo Close™ device introduced via a 2–3 mm skin incision for retrieval of suture ends. **D** peritoneal entry must be judged to ensure creation of a 1 cm^2^ box configuration which mirrors that at the jejunum. **E** Jejunostomy insertion trocar targeted at centre of jejunal box target. **F** Jejunostomy tube delivered via trocar, with visual confirmation of placement towards distal limb, and saline flush of tube for confirmation of patency and positioning. **G** Antirotation suture placed at nearby distal jejunal limb

Laparoscopic jejunostomy insertion was performed using a three-port technique modified from Senkal and colleagues (Fig. 1. An additional movie file demonstrates the technique in further detail, see Additional file 1) [9]. Briefly, a 12 mm laparoscopic port was placed in the right flank to accommodate the laparoscope, and a further two 5 mm working ports were inserted under vision. The DJ-flexure was identified, and a loop of proximal jejunum confirmed to reach the abdominal wall with ease was selected. Placement of two absorbable braided sutures in a ‘W’ configuration was performed to define a square area that would become the jejunostomy insertion point (Fig. 1B). The four ends of the two sutures were retrieved outside the abdominal cavity through a 2–3 mm skin incision using Endo Close™ Trocar Site Closure Device (Covidien, USA), and ensuring that the points of retrieval or exit from the peritoneum are adequately spaced to mirror the square area created by the sutures placed at the jejunum (Fig. 1D). The jejunostomy kit’s introducer is inserted through the abdominal wall into the jejunum, thereby permitting direct passing of the tube distally. Positioning is visually verified and tested by saline infusion, with vigorous flushing alongside gentle laparoscopic manipulation employed to promote uncoiling where possible. The intra-abdominal pressure is lowered to 5 mmHg to enable parachuting of the small bowel up to the abdominal wall upon tying of the extracorporeal sutures. The procedure is completed by securing the jejunostomy tube using the flange supplied as per the open approach aforementioned.

### Statistical analyses

Statistical analyses were performed using GraphPad Prism v7.0 Software. Group comparisons of continuous variables were performed by t test, where normality was confirmed using the D’Agostino-Pearson, or by Mann–Whitney U test where not normally distributed. Chi-square or Fisher’s-exact test, when appropriate, were used to compare categorical variables. A P value of less than 0.05 was considered significant.

## Results

After performing a database search, 175 consecutive jejunostomy procedures were identified during the study period, 157 of which were associated with the management of oesophageal malignancy and therefore eligible for inclusion. Of this cohort, 126 subjects underwent open jejunostomy insertion while 31 underwent laparoscopic insertion. Comparisons of subject characteristics between both groups are shown in Table 1, and operative approaches to resectional procedures are outlined in Table 2. While groups were comparable for sex, body mass index (BMI), serum albumin, pre-procedural performance status, tumour histology, and tumour stage, the laparoscopic group were marginally younger than those undergoing open surgery (P = 0.014).

**Table 1:** Comparison of preoperative demographic and nutritional variables. *n* (%)

	Open jejunostomy (n = 126)	Laparoscopic jejunostomy (n = 31)	P value
Age at surgery (years)	66.9	62.6	0.014
Sex is ‘man’ n (%)	99 (79)	21 (68)	0.20
Mean Body Mass Index (kg/m^2^)	27.9	26.7	0.36
Mean preoperative serum albumin (g/L)	32.3	31.0	0.20
Median performance status (ECOG)	0	0	0.95
TNM			
T 1	7 (6)	4 (13)	NS
2	27 (23)	8 (27)	
3	76 (63)	15 (50)	
4	10 (8)	3 (10)	
N 0	43 (35)	14 (47)	NS
1	54 (44)	13 (43)	
2	22 (18)	3 (10)	
3	4 (3)	0 (-)	
Histology group			
Squamous cell carcinoma	24 (19)	7 (23)	NS
Adenocarcinoma	100 (79)	23 (74)	
Other	2 (2)	1 (3)	
ASA Classification			
I	32 (25)	12 (40)	NS
II	89 (71)	18 (60)	
III	6 (4)	0 (-)	
Comorbidities			
None	61	17	
Active Smoker	11	8	
Asthma/COPD	8	5	NS
Hypertension	30	3	
Coronary artery disease	9	0	
History of stroke	0	1	
Diabetes mellitus	13	3	
Prior abdominal surgery	5	3

**Table 2:** Surgical approach undertaken among resectional procedures performed

	N (%)
Surgical approach	
Left-thoracoabdominal	41 (47)
Ivor-Lewis	21 (24)
Hybrid Ivor-Lewis (Laparoscopic)	8 (9)
Minimally invasive	15 (17)
3-Stage	1 (1)

Comparisons of operative outcomes are shown in Table 3. Jejunostomy insertion was performed as a stand-alone procedure or in conjunction with a laparoscopic staging procedure (SP), which is to say prior to definitive oesophagectomy, in 71 (45.2%) of 157 cases. The remaining 86 (54.8%) cases underwent insertion at the time of tumour resection procedure (RP). A trend towards a higher incidence of complications was noted among those in the ‘staging’ (SP) group (29/71, 40.8%) as compared with the RP group (25/86, 29.1%), though this did not reach statistical significance (P = 0.12). The rate of *major* complications (CD ≥ 3) however, was noted to be significantly higher at SP (P = 0.011), a finding predominantly attributable to the particularly high rates of complications seen in cases of open insertion in this setting (11/63, 17.5%, P = 0.016).

**Table 3:** Comparison of surgical outcomes among patients with oesophagogastric cancer undergoing laparoscopic or open insertion of feeding jejunostomy either at the time of tumour resection procedure (RP) or at a stand-alone/staging procedure (SP)

	At Staging Procedure (SP)		P	At Resection Procedure (RP)		P
	Open (n = 63)	Laparoscopic (n = 8)		Open (n = 63)	Laparoscopic (n = 23)	
Jejunostomy-related complications						
Minor complications (CD ≤ 2)	16 (25.4%)	2 (25.0%)	0.99	19 (30.2%)	3 (13.0%)	0.16
Dislodgement	4	–		1	–	
Leakage	–	–		2	1	
Infection	5	1		5	0	
Ileus	–	–		1	–	
Feed related symptoms (diarrhoea, distension, pain)	7	1		10	2	
(All SP vs. all RP in minor category: P = 0.97)						
Major complications (CD ≥ 3)	11 (17.5%)	0 (0%)	0.34	2 (3.2%)	1 (4.3%)	0.99
Dislodgement (with RT/IR)	2	–		1	–	
Occlusion (with RT/IR)	2	–		1	–	
Peritonitis/leak	2	–		–	–	
Wound infection/dehiscence	1	–		–	–	
Bowel obstruction	4	–		–	1	
(All SP vs. all RP in major category: P = 0.011)						
Total jejunostomy related complications	27 (42.9%)	2 (25%)	0.46	21 (33.3%)	4 (17.4%)	0.19
(All SP vs. all RP: P = 0.12) (All Open vs. all Laparoscopic: P = 0.049)						
Clavien-Dindo Classification of jejunostomy related complications						
Grade I	9	1	0.60	13	2	0.34
Grade II	7	1	0.27	6	1	0.67
Grade IIIa	3	–	0.99	–	1	0.27
Grade IIIb	8	–	0.58	2	–	0.99
Grade IV	–	–	–	–	–	–
Grade V	1	–	0.99	–	–	–
Resection (non-jejunostomy) complications						
Anastomotic leak				2	1	
Pneumonia				21	6	
Arrhythmia				7	1	
Chyle leak				4	6	
Return to theatre				1	–	
Thromboembolism				2	–	
Myocardial infarction				1	–	
Median length of stay (Days)	3	1.5	0.17	10	9	0.09

*n* (%). *RT* return to theatre, *IR* invasive procedure by interventional radiology

Across all procedures performed, significantly fewer complications were encountered following laparoscopic insertion as compared with open (P = 0.049). Among the subset of cases undergoing jejunostomy insertion at SP, a substantially greater proportion of those performed open encountered major complications (11, 17.5%), than did those performed laparoscopically (0, 0%), though not reaching statistical significance (P = 0.34). Similarly, in the RP group, 21 major or minor complications were encountered among the 63 patients undergoing open jejunostomy placement (33.3%), whilst 4 (17.3%) were noted among the laparoscopic group (P = 0.19). In this setting however, although only jejunostomy-associated complications were analysed, the impact of the resectional approach itself is not controlled for. Median length of stay was longer amongst those undergoing open procedures in both the SP (1.5 vs. 3 days, P = 0.17) and the RP (9 vs. 10, P = 0.09).

With regards to the small but significant difference of approximately 4 years in mean age among groups, increasing age was not noted to be associated with higher rates of complications (P = 0.10). One death occurred in the cohort of 157 cases (0.6%), and this was following open jejunostomy insertion at the time of staging. The death was associated with the perforation of a closed bowel loop involving a segment between the obstructive oesophageal tumour and the jejunostomy site.

## Discussion

The integration of appropriate nutritional support into the overall management of patients undergoing curative treatment for cancer of the oesophagus is of utmost importance to the successful completion of neoadjuvant therapy and of surgery, as well as to survival outcomes [10, 11]. Feeding jejunostomy is the preferred approach to long-term enteral feeding and, since minimally invasive techniques have many advantages, total laparoscopic or laparoscopically assisted methods of feeding jejunostomy insertion have garnered increasing attention [12].

This study, which represents the largest to our knowledge comparison of total laparoscopic and open jejunostomy insertion in the setting of oesophageal cancer, shows that laparoscopic feeding jejunostomy can be performed safely and has the potential to confer the benefits of minimally invasive surgery including lower rates of morbidity and shorter hospitalisations. Furthermore, the study highlights the risk of major complications among subjects undergoing jejunostomy insertion as an open stand-alone procedure. This finding is in keeping with the literature, wherein reported complication rates for this approach range widely but can reach 37% and beyond in some series [12, 13]. One explanation for the higher rates of complication encountered in stand-alone open insertions is that such procedures are generally performed as ‘mini-laparotomies’ to avoid the morbidity of larger incisions. As a consequence, they may not afford sufficient exposure and access to ensure adequate visualisation of the DJ-flexure, jejunum, and abdominal wall. Suboptimal exposure can lead to inadvertent injury, kinking or narrowing of the lumen, and difficulties with fixation to the abdominal wall. The laparoscopic technique described here enabled excellent visualisation of the DJ-flexure and of the abdominal wall, and the use of the Endo Close™ device makes parachuting the jejunum to the abdominal wall uncomplicated. The direct puncture of the jejunum, without tunnelling, was a source of concern at the beginning of our experience, but leakage of enteric content was encountered in only one case and was successfully managed with antibiotics and temporary suspension of enteral feeding. A second consideration is that open procedures may be performed by less experienced junior surgeons as compared with laparoscopic approaches. In our series, the number of laparoscopic jejunostomy insertions was relatively lower (31 vs. 126 open) due to the fact that only one senior surgeon in our unit employs the laparoscopic technique and, as a consequence, all such procedures were either performed by this senior surgeon or by a junior surgeon under direct supervision. In addition to this latter point, this study is limited by the small number, specifically in the subgroup of procedures performed laparoscopically as a staging or stand-alone procedure.

The postoperative mortality in our series of 157 patients was 0.6%, again mirroring published reports. The single death involved a subject with an obstructing tumour at the gastro-oesophageal junction, a closed-loop obstruction which was diagnosed late, and multiple comorbidities. While mortality rates are low, the significant risks of minor or major complications highlight the value of careful patient selection pre-operatively, emphasise the importance of patient counselling and informed consent, and focus attention on the development of improved surgical approaches.

The cost-effectiveness of laparoscopic surgery is often questioned. Though not formally assessed in our study, in broad terms the cost of the equipment and length of operation time should be balanced against potential benefits regarding the length of stay, cosmesis, reduced analgesic requirements, and improved outcomes. It is important to note, that laparoscopic jejunostomy insertion was predominantly performed in conjunction with either staging laparoscopy or with minimally invasive oesophagectomy, and thereby incurred no significant additional equipment costs. This study is limited by its retrospective nature and in that data is derived from a single centre. Though this represents the largest comparative study of total laparoscopic and open jejunostomy insertion in the setting of oesophageal cancer, the nevertheless is limited by its relatively small patient population. Future prospective studies comparing laparoscopic vs. open feeding jejunostomy insertion are required to determine the superiority of either approach. Our report focuses on patients undergoing treatment for oesophageal malignancy and, however likely, it remains to be confirmed whether the findings hold true in patients undergoing jejunostomy insertion in the contexts of gastric, pancreatic, or hepatic malignancies for instance.

## Conclusions

Feeding jejunostomy represents a key adjunct in the treatment of oesophageal malignancy. Though considered by some to be a routine, innocuous procedure, open insertion of feeding jejunostomy is a procedure which should not be underestimated. Keeping in mind the aims of successful neoadjuvant treatment and the reduction of early postoperative morbidity, any complications arising from such a procedure could jeopardise potential benefits and incur significant costs. The total laparoscopic approach outlined here can serve as a safe, effective alternative with the associated advantages of minimally invasive surgery.

## Supplementary Information

**Additional file 1. **Intraoperative video demonstration of laparoscopic feeding jejunostomy placement and positioning.

## Data Availability

The datasets generated and/or analysed during the current study are not publicly available due to patient data confidentiality but are available from corresponding author upon reasonable request.

## Abbreviations

BMI: Body mass index
CD: Clavien-Dindo Score
DJ: Duodenojejunal
SP: Staging/stand-alone procedure
RP: Resection procedure
